# Periostin expression in intra-tumoral stromal cells is prognostic and predictive for colorectal carcinoma *via* creating a cancer-supportive niche

**DOI:** 10.18632/oncotarget.5985

**Published:** 2015-11-09

**Authors:** Xiaowen Xu, Wenjun Chang, Jie Yuan, Xue Han, Xiaojie Tan, Yibo Ding, Yanxin Luo, Hui Cai, Yan Liu, Xianhua Gao, Qizhi Liu, Yongwei Yu, Yan Du, Hao Wang, Liye Ma, Jianping Wang, Kun Chen, Yanqing Ding, Chuangang Fu, Guangwen Cao

**Affiliations:** ^1^ Department of Colorectal Surgery, The 1^st^ Affilaited Hospital, Second Military Medical University, Shanghai, China; ^2^ Department of Epidemiology, Second Military Medical University, Shanghai, China; ^3^ Department of Chronic Diseases, Center for Diseases Control and Prevention of Yangpu District, Shanghai, China; ^4^ Department of Colorectal Surgery, The Sixth Affiliated Hospital, Sun Yat-sen University, Guangzhou, China; ^5^ Department of Pathology, The 1^st^ Affilaited Hospital, Second Military Medical University, Shanghai, China; ^6^ Department of General Surgery, The 1^st^ Affilaited Hospital, Second Military Medical University, Shanghai, China; ^7^ Department of Epidemiology and Biostatistics, Zhejiang University School of Public Health, Hangzhou, China; ^8^ Department of Pathology, Nanfang Hospital, Southern Medical University, Guangzhou, China

**Keywords:** colorectal carcinoma, periostin, fibroblast, drug-resistance, prognosis

## Abstract

Periostin (POSTN) expression in cancer cells and circulation has been related to poor prognosis of colorectal carcinoma (CRC). However, the role of POSTN expressed in intra-tumoral stroma on CRC progression remains largely unknown. This study enrolled 1098 CRC patients who received surgical treatment in Shanghai and Guangzhou, Mainland China. In Shanghai cohort, immunohistochemistry score of stromal POSTN expression increased consecutively from adjacent mucosa, primary CRC tissues, to metastatic CRC tissues (*P* < 0.001), while medium- and high-stromal POSTN expression, rather than epithelial POSTN expression, independently predicted unfavorable prognoses of CRC, adjusted for covariates including TNM stage and postoperative chemotherapy in multivariate Cox models. The results in Shanghai cohort were faithfully replicated in Guangzhou cohort. Stromal POSTN expression dose-dependently predicted an unfavorable prognosis of stage III CRC patients with postoperative chemotherapy in both cohorts. POSTN derived from colonic fibroblasts or recombinant POSTN significantly promoted proliferation, anchorage independent growth, invasion, and chemo-resistance of CRC cells; whereas these effects were counteracted *via* targeting to PI3K/Akt or Wnt/β-catenin signaling pathway. CRC cell RKO-derived factor(s) significantly induced POSTN production in colonic fibroblasts and autocrine POSTN promoted proliferation, migration, and anchorage independent growth of fibroblasts. Conclusively, stromal POSTN is prognostic and predictive for CRC *via* creating a niche to facilitate cancer progression. Targeting POSTN-induced signaling pathways may be therapeutic options for metastatic or chemoresistant CRC.

## INTRODUCTION

Colorectal cancer (CRC) is one of the most common malignancies worldwide, with 1.23 million newly diagnosed cases annually [1]. Surgical resection is the primary option for CRC treatment. Approximately 20%–25% of CRC patients present with metastatic disease at diagnosis and another 20%–25% will develop metachronous metastases, resulting in an overall mortality rate of 40%–45% [2]. Tumor stage, circumferential resection margin involvement, obstruction/perforation, rupture during surgery, vascular and perineural invasion, poor differentiation, high-frequency microsatellite instability, mismatch repair deficiency, thymidylate synthase positivity, circulating tumor cells, KRAS mutation, BRAF mutation, and elevated expression of Snail or serpinA1 have been suggested to be prognostic for CRC [2–5]. Postoperative chemotherapy is the standard of care for stage III CRC patients. Some molecules or signatures expressed in CRC tissues can be predictive [6, 7]. Although these molecules are potentially prognostic and/or predictive, few of them are clinically applied. Identification of new biomarkers to improve CRC prognosis classification and individualized treatment is urgently needed.

Previously, we have demonstrated that periostin (POSTN) is the top overlapping molecule in signatures (4/5) with high concordance and repeatable prognostic values for CRC. Further immunohistochemistry (IHC) assays have demonstrated that POSTN is a key component of the CRC cell-derived gene signature with robust prognostic and predictive values [8]. POSTN, firstly identified as osteoblast-specific factor 2 in 1993, is a secretory, matricellular molecule with known functions in bone development/maturation/repair, tissue repair, mesenchymal remodeling, and epithelial-mesenchymal transition (EMT) [9]. POSTN expression in cancer cells (epithelial POSTN expression) can promote metastatic potential of CRC, *via* activating the PI3 kinase (PI3K)/protein kinase B (Akt) signaling pathway [10]. In CRC with liver metastasis, POSTN is significantly higher in CD133^+^ than in CD133^−^ tumor cells [11]. We and others observed that POSTN was highly expressed in stromal cells in CRC tissues [8, 12]. POSTN expressed in cancer-associated fibroblasts or other stromal cells may facilitate the aggressiveness of pancreatic cancer, ovarian cancer, prostate cancer, esophageal adenocarcinoma, gastric cancer, breast cancer, and cholangiocarcinoma [13–19]. The elevated level of POSTN in sera is also associated with an unfavorable prognosis of CRC [20]. However, the effect of POSTN expression in intratumoral stromal cells (stromal POSTN expression) on the progression and prognosis of CRC remains largely unknown. The objective of this study is to clarify if stromal POSTN expression in tumor tissues is prognostic and/or predictive for CRC and elucidate the mechanisms by which stromal POSTN promoted the aggressiveness and drug-resistance of CRC. This study should be helpful for the prediction and targeted treatment of metastatic or chemoresistant CRC.

## RESULTS

### Expression pattern of POSTN in adjacent mucosa, primary CRC, and metastatic CRC tissues

**Figure 1:**
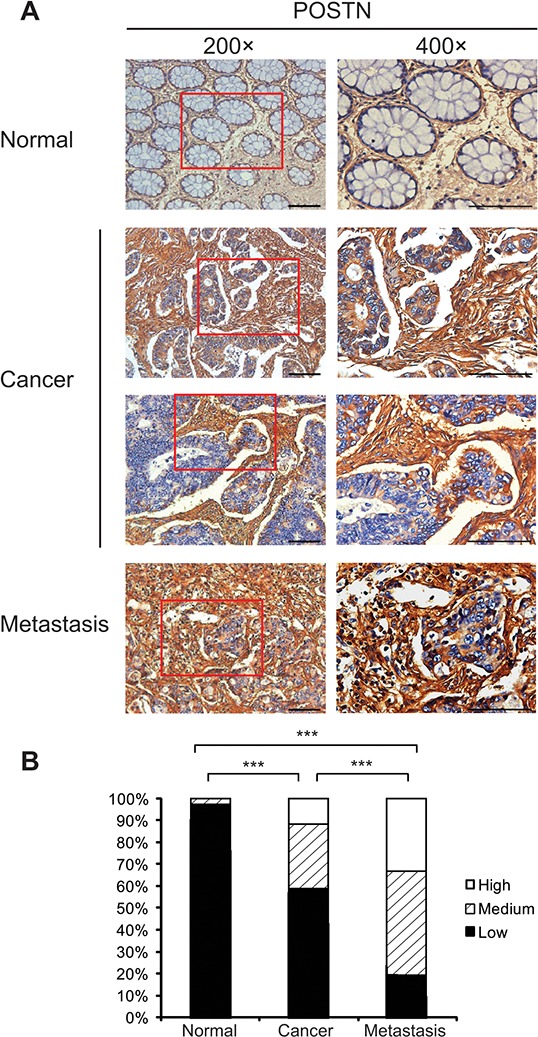
Expression pattern of POSTN in formalin-fixed paraffin-embedded specimens of adjacent pathologically normal mucosa, primary tumors, and metastatic tumors of CRC patients in Shanghai cohort **A.** Representative immunostainings of POSTN in adjacent mucosa tissues, primary tumors, and metastatic tumors. POSTN was expressed in the cytoplasm of epithelial cells and stromal cells. Bar, 50 μm. **B.** Expression pattern of POSTN protein in adjacent mucosa tissues, primary tumors, and metastatic tumors. Abbreviation and mark: CRC, colorectal carcinoma; ***, *P* < 0.001.

Tissue microarrarys (TMAs) containing surgically removed adjacent mucosa (*n* = 37), primary CRC at I–IV stages (*n* = 755), and metastatic CRC specimens (*n* = 21) from the 1^st^ Affiliated Hospital of Second Military Medical University were immunostained using antibody against POSTN. POSTN was mainly expressed in the cytoplasm of stromal cells and epithelial CRC cells, as shown in Figure 1A. The immunostaining of POSTN was more intensive in stromal cells than in epithelial cancer cells in the specimens of 506 (67.02%) of the 755 CRC patients. IHC score of stromal POSTN expression was significantly higher in the metastatic CRC tissues than in primary CRC tissues (*P* < 0.001) and also significantly higher in primary CRC tissues than in the adjacent mucosa (*P* < 0.001), as shown in Figure 1B. According to IHC scores of stromal or epithelial POSTN expression, CRC patients were classified into 3 groups: low- (0–4), medium- (6–8), and high-score (9–12) groups. High IHC score of stromal POSTN expression was significantly associated with low differentiation grade (*P* < 0.001) and high TNM stage (*P* < 0.001) in 755 CRC patients (Table 1).

**Table 1:** Associations of stromal POSTN expression with demographic and clinical variables of 755 CRC patients in Shanghai cohort

Characteristics	Stromal POSTN expression in tumor specimens			
	Low (*n* = 444)	Medium (*n* = 222)	High (*n* = 89)	*P* value
Age (years), mean (SD)	60.33 (12.77)	58.96 (13.23)	57.61 (11.97)	0.133
Sex, *n* (%)				
Women	193 (43.5)	90 (40.5)	37 (41.6)	0.761
Men	251 (56.5)	132 (59.5)	52 (58.4)	
Disease location, *n* (%)				
Colon	210 (47.3)	102 (45.9)	42 (47.2)	0.945
Rectum	234 (52.7)	120 (54.1)	47 (52.8)	
Differentiation grade, *n* (%)				<0.001
Well	16 (3.6)	8 (3.6)	1 (1.1)	
Moderately	269 (60.6)	119 (53.6)	36 (40.4)	
Poorly	125 (28.2)	79 (35.6)	49 (55.1)	
Missing	34 (7.7)	16(7.2)	3(3.4)	
Number of lymph nodes, *n* (%)				0.607
<12	198 (44.6)	96 (43.2)	44 (49.4)	
≥12	246 (55.4)	126 (56.8)	45 (50.6)	
TNM stage, *n* (%)				<0.001
I	40 (9.0)	10 (4.5)	2 (2.2)	
II	193 (43.5)	94 (42.3)	24 (27.0)	
III	190 (42.8)	87 (39.2)	42 (47.2)	
IV	21 (4.7)	31 (14.0)	21 (23.6)	
Adjuvant chemotherapy, *n* (%)				<0.001
Yes	362 (81.5)	199 (89.6)	86 (96.6)	
No	82 (18.5)	23 (10.4)	3 (3.4)	
Serum CEA				
<5 ng/mL	283 (63.7)	133 (59.9)	50 (56.2)	0.374
≥5 ng/mL	161 (36.3)	89 (40.1)	38 (42.7)	
Missing	0 (0)	0 (0)	1 (1.1)	
Serum CA19-9				
<37 U/ml	379 (85.4)	180 (81.1)	68 (76.4)	0.123
≥37 U/ml	65 (14.6)	41 (18.5)	20 (22.5)	
Missing	0 (0)	1 (0.5)	1 (1.1)	

a Student-Newman-Keuls *q* test

b Pearson Chi-Square test

c Kruskal-Wallis test.

### Stromal POSTN expression had higher discriminatory performances than epithelial POSTN expression in predicting postoperative prognosis of CRC

**Figure 2:**
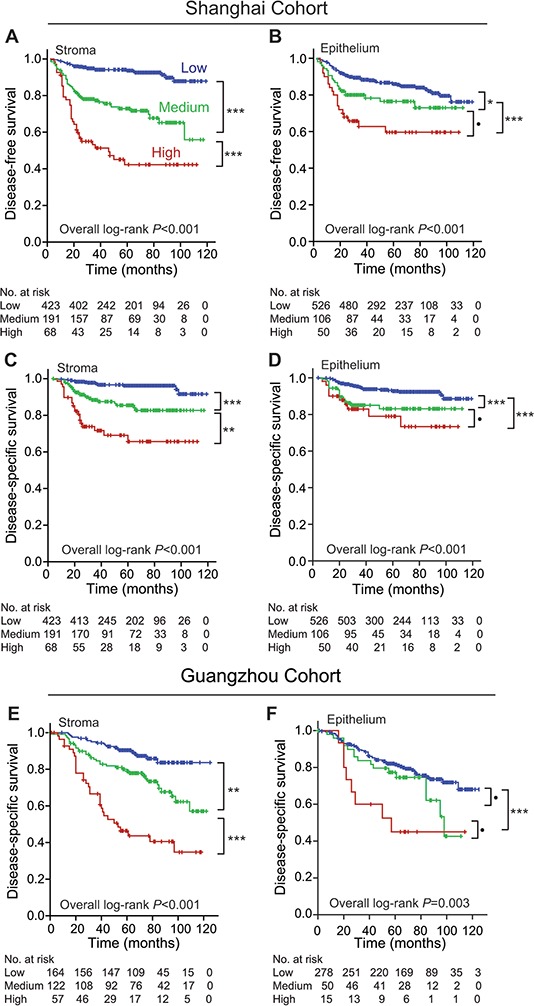
Comparison of discriminatory performances between stromal and epithelial POSTN expression in predicting postoperative prognoses of stage I–III CRC patients in both cohorts **A.** stromal POSTN expression predicted DFS of CRC patients in Shanghai cohort. **B.** epithelial POSTN expression predicted DFS of CRC patients in Shanghai cohort. **C.** stromal POSTN expression predicted DSS of CRC patients in Shanghai cohort. **D.** epithelial POSTN expression predicted DSS of CRC patients in Shanghai cohort. **E.** stromal POSTN expression predicted DSS of CRC patients in Guangzhou cohort. **F.** epithelial POSTN expression predicted DSS of CRC patients in Guangzhou cohort. Abbreviations and marks: CRC, colorectal carcinoma; DFS, disease-free survival; DSS, disease-specific survival; •, *P* > 0.05; *, *P* < 0.05; **, *P* < 0.01; ***, *P* < 0.001.

To compare the contributions of stromal POSTN and epithelial POSTN expression to postoperative prognosis of CRC, we initially included 682 stage I-III CRC patients who received curative surgery and regular follow-up in Shanghai cohort. The 5-year rates of disease-free survival (DFS) were 93.6%, 72.9%, and 42.3%; while the 5-year rates of disease-specific survival (DSS) were 96.2%, 85.4%, and 65.6%, in the groups with low-, medium-, and high-score of stromal POSTN expression, respectively (*P* < 0.001 for each). Patients with higher score of stromal POSTN expression had shorter DSS and DFS (Figure 2A, 2C). In terms of epithelial POSTN expression, however, the survivals were not significantly different between patients with high-score and those with medium-score (Figure 2B, 2D). Next, the prognostic values of POSTN expression were validated in Guangzhou cohort with 343 CRC patients who received curative surgery and regular follow-up. The associations of POSTN expression levels in stromal cells and in cancer cells with DSS observed in Shanghai cohort were faithfully replicated in Guangzhou cohort, as shown in Figure 2E, 2F.

Multivaritate Cox regression analyses showed that medium-score and high-score stromal POSTN expression, rather than epithelial POSTN expression, were independent risk factors for DFS and DSS in Shanghai cohort, adjusted for the covariates including TNM stage, tumor differentiation grade, and postoperative chemotherapy; higher stromal POSTN expression also predicted a poorer DSS in Guangzhou cohort independently, as shown in Table 2.

**Table 2:** Contribution of stromal POSTN expression, epithelial POSTN expression, and demographic and clinicopathological covariates to postoperative survivals of CRC patients in multivariate Cox hazard proportion models

	Shanghai Cohort				Guangzhou Cohort	
	DFS		DSS		DSS	
	HR (95% CI)	*P* value	HR (95% CI)	*P* value	HR (95% CI)	*P* value
Stromal POSTN score						
Low	1.00		1.00		1.00	
Medium	5.14 (3.21–8.23)	<0.001	4.14 (2.12–8.11)	<0.001	2.71 (1.43–5.14)	0.002
High	11.85 (6.61–21.23)	<0.001	9.18 (4.15–20.34)	<0.001	5.69 (2.93–11.05)	<0.001
Epithelial POSTN score						
Low	1.00		1.00		1.00	
Medium	0.63 (0.38–1.04)	0.069	1.06 (0.55–2.04)	0.868	1.22 (0.64–2.32)	0.541
High	0.83 (0.46–1.50)	0.534	1.16 (0.51–2.62)	0.720	3.76 (1.50–9.41)	0.005
TNM stage (III vs I + II)	1.86 (1.20–2.88)	0.006	1.53 (0.84–2.80)	0.167	1.39 (0.84–2.32)	0.204
Differentiation grade (poorly vs well + moderately)	1.11 (0.75–1.65)	0.614	1.03 (0.559–1.79)	0.929	1.56 (0.68–3.57)	0.294
Adjuvant chemotherapy (yes vs no)	1.79 (0.74–4.36)	0.200	1.45 (0.48–4.39)	0.516	1.00 (0.59–1.72)	0.987
Age (≥50 vs <50 years)	0.82 (0.54–1.25)	0.36	1.39 (0.72–2.70)	0.332	1.41 (0.79–2.50)	0.247
Sex (male vs female)	1.19 (0.81–1.73)	0.378	1.27 (0.74–2.18)	0.382	1.04 (0.63–1.71)	0.877
Disease location (rectum vs colon)	1.18 (0.81–1.71)	0.395	1.35 (0.80–2.27)	0.257	1.58 (0.91–2.74)	0.104
Lymph nodes examined (≥12 vs <12)	2.22 (1.47–3.33)	<0.001	1.96 (1.12–3.45)	0.019	0.94 (0.58–1.55)	0.819
Serum CEA (ng/ml) (≥5 vs <5)	0.99 (0.65–1.52)	0.991	1.02 (0.57–1.83)	0.937	1.51 (0.90–2.52)	0.119
Serum CA19–9 (U/ml) (≥37 vs <37)	1.52 (0.93–2.51)	0.098	1.42 (0.71–2.82)	0.324	2.02 (1.14–3.58)	0.015

Abbreviations: CRC, colorectal carcinoma; DFS, disease-free survival; DSS, disease-specific survival; HR, hazard ratio; CI, confidence interval; CEA, carcinoembryonic antigen; IHC, immunohistochemistry.

### Stromal POSTN expression predicted the prognosis of CRC patients who received postoperative chemotherapy

**Figure 3:**
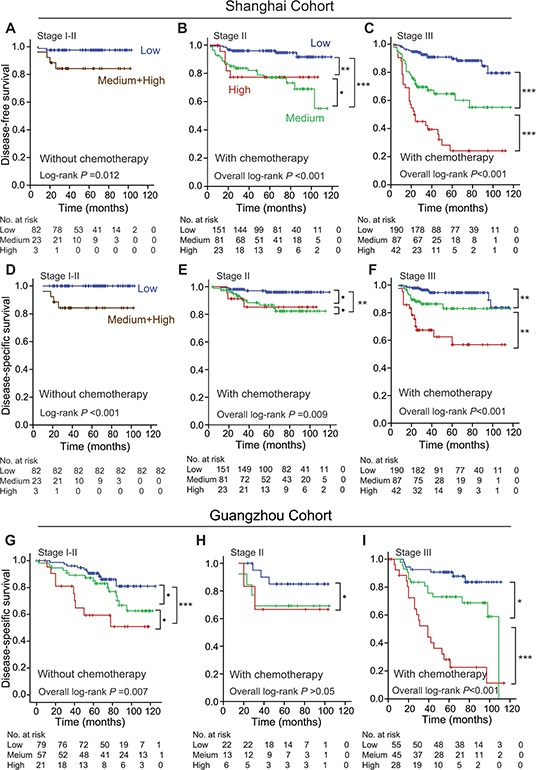
Effects of stromal POSTN expression in predicting postoperative prognoses of CRC patients with or without postoperative chemotherapy in both cohorts **A.** DFS of stage I-II CRC patients without chemotherapy in Shanghai cohort. **B.** DFS of stage II CRC patients with postoperative chemotherapy in Shanghai cohort. **C.** DFS of stage III CRC patients with postoperative chemotherapy in Shanghai cohort. **D.** DSS of stage I-II CRC patients without chemotherapy in Shanghai cohort. **E.** DSS of stage II CRC patients with postoperative chemotherapy in Shanghai cohort. **F.** DSS of stage III CRC patients with postoperative chemotherapy in Shanghai cohort. **G.** DSS of stage I-II CRC patients without chemotherapy in Guangzhou cohort. **H.** DSS of stage II CRC patients with postoperative chemotherapy in Guangzhou cohort. **I.** DSS of stage III CRC patients with chemotherapy in Guangzhou cohort. Abbreviations and marks: CRC, colorectal carcinoma; DFS, disease-free survival; DSS, disease-specific survival; •, *P* > 0.05; *, *P* < 0.05; **, *P* < 0.01; ***, *P* < 0.001.

We stratified patients into stage I-II CRC without chemotherapy, stage II CRC with postoperative chemotherapy, and stage III CRC with postoperative chemotherapy in Shanghai cohort. It was found that stromal POSTN expression had higher discriminatory performances of predicting DFS and DSS in stage III CRC patients with postoperative chemotherapy than in stage I-II patients without chemotherapy and in stage II patients with postoperative chemotherapy. The result of DSS was consistently replicated in Guangzhou cohort. The results are shown in Figure 3.

### POSTN promoted proliferation, anchorage independent growth, and invasion of CRC cells

**Figure 4:**
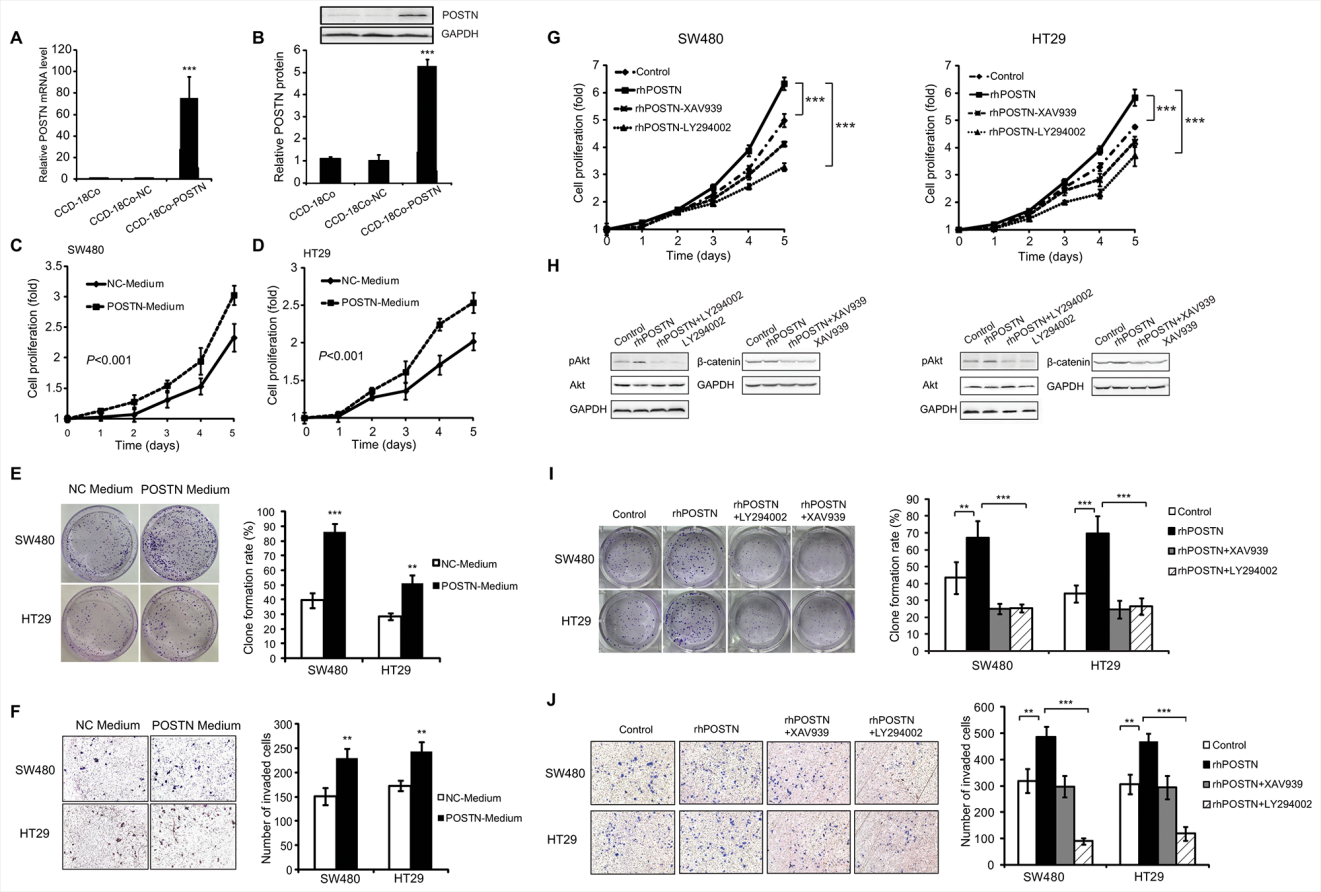
Effects of POSTN on proliferation, anchorage independent growth, and invasion of CRC cells and its contributing signaling pathways **A.** POSTN mRNA levels in CCD-18Co-POSTN, CCD-18Co-NC, and CCD-18Co cells. **B.** POSTN protein levels in CCD-18Co-POSTN, CCD-18Co-NC, and CCD-18Co cells. **C.** proliferation of SW480 cells in CCD-18Co-POSTN medium. **D.** proliferation of HT29 cells in CCD-18Co-POSTN medium. **E.** anchorage independent growth of CRC cells in CCD-18Co-POSTN medium. **F.** invasion of CRC cells in CCD-18Co-POSTN medium. **G.** proliferation of CRC cells treated with 100 ng/mL rhPOSTN, rhPOSTN plus 10 μM PI3K/Akt kinase inhibitor LY294002, and rhPOSTN plus 10 μM β-catenin-specific inhibitor XAV939. **H.** expression levels of pAkt and β-catenin in CRC cells treated with POSTN and/or signaling inhibitors by Western blot analyses. **I.** anchorage independent growth of CRC cells treated with POSTN and/or signaling inhibitors. **J.** invasion of CRC cells treated with POSTN and/or signaling inhibitors. Abbreviations and marks: CRC, colorectal carcinoma; pAkt, phosphorylation of Akt; *, *P* < 0.05; **, *P* < 0.01; ***, *P* < 0.001.

We constructed a recombinant lentiviral vector containing a full-length of human POSTN cDNA. After being amplified in 293T cells, the resulting recombinant lentivirues were employed to infect CCD-18Co, a colon fibroblast cell line, and generated a stable POSTN-expression cell line named CCD-18Co-POSTN. Similarly, the corresponding empty lentiviral vector was applied to generate a control fibroblast clone named CCD-18Co-NC. With the use of quantitative reverse transcription PCR (qRT-PCR) and Western blot, we confirmed that POSTN expression was significantly higher in CCD-18Co-POSTN than in CCD-18Co-NC and parental CCD-18Co cells, respectively (Figure 4A, 4B). To elucidate the effects of POSTN derived from fibroblasts on CRC cells, we examined proliferation, anchorage independent growth, and invasion of SW480 and HT29 cells cultured with conditional media of CCD-18Co-POSTN and CCD-18Co-NC cells, respectively. As expected, SW480 and HT29 cells proliferated faster and formed more clones in CCD-18Co-POSTN medium than in CCD-18Co-NC medium (Figure 4C, 4D, 4E). CCD-18Co-POSTN medium significantly enhanced invasion of SW480 and HT29 cells than did CCD-18Co-NC medium (Figure 4F). To further elucidate the role of POSTN on the aggressiveness of CRC cells and the underlying mechanism, recombinant human POSTN (rhPOSTN), PI3K/Akt kinase inhibitor LY294002, and β-catenin-specific inhibitor XAV939 were applied in the *in vitro* study of CRC cells. It was found that 100 ng/mL rhPOSTN significantly increased proliferation of SW480 and HT29 cells; whereas 10 μM LY294002 or XAV939 significantly decreased proliferation of CRC cells (Figure 4G). Western blot assays showed that rhPOSTN apparently increased phosphorylation of Akt (pAkt) in SW480 and HT29 cells and expression level of β-catenin in HT29 cells; whereas 10 μM LY294002 or XAV939 apparently reduced rhPOSTN-upregulated expression of pAkt and β-catenin in CRC cells (Figure 4H). Anchorage independent growth of CRC cells was significantly increased by rhPOSTN; however, this effect was significantly counteracted by LY294002 or XAV939 (Figure 4I). Invasion of CRC cells was also significantly increased by rhPOSTN but greatly reduced by the inhibitors, especially by PI3K/Akt kinase inhibitor (Figure 4J).

### POSTN increased the chemo-resistance of CRC cells *via* activating PI3K/Akt and/or Wnt/β-catenin signaling pathways

**Figure 5:**
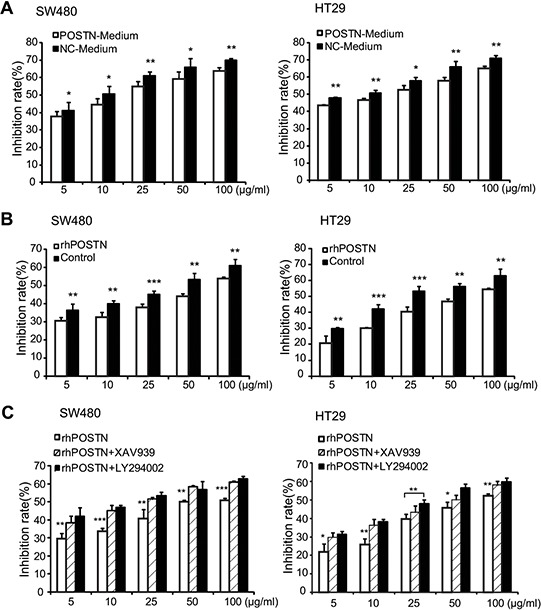
Chemo-resistance of CRC cells treated with POSTN and its contributing signaling pathways **A.** inhibitory effect of 5-FU on proliferation of CRC cells cultured with CCD-18Co-POSTN medium or CCD-18Co-NC medium. **B.** inhibitory effect of 5-FU on proliferation of CRC cells cultured with 100 ng/ml rhPOSTN. **C.** rhPOSTN-upregulated chemo-resistance of CRC cells were significantly decreased by 10 μM PI3K/Akt kinase inhibitor LY294002 or β-catenin-specific inhibitor XAV939. Abbreviations and marks: CRC, colorectal carcinoma; 5-FU, 5-fluorouracil; rhPOSTN, human recombinant POSTN; *, *P* < 0.05; **, *P* < 0.01; ***, *P* < 0.001.

Conditional medium of CCD-18Co-POSTN cells significantly increased the chemo-resistance of SW480 and HT29 cells to 5-fluorouracil (5-FU), compared to counterparts cultured with CCD-18Co-NC medium (Figure 5A). Further experimentation indicated that 100 ng/mL rhPOSTN greatly increased the chemo-resistance of both CRC cells to 5-FU (Figure 5B). Interestingly, the rhPOSTN-increased chemo-resistance of SW480 cells was significantly decreased when the signaling inhibitor LY294002 or XAV939 was present in the culture; the chemo-resistance of HT29 was also decreased by the inhibitors, especially by PI3K/Akt kinase inhibitor LY294002 (Figure 5C).

### Stromal POSTN promoted proliferation, migration, and anchorage independent growth of fibroblasts *via* paracrine or autocrine modes of action

**Figure 6:**
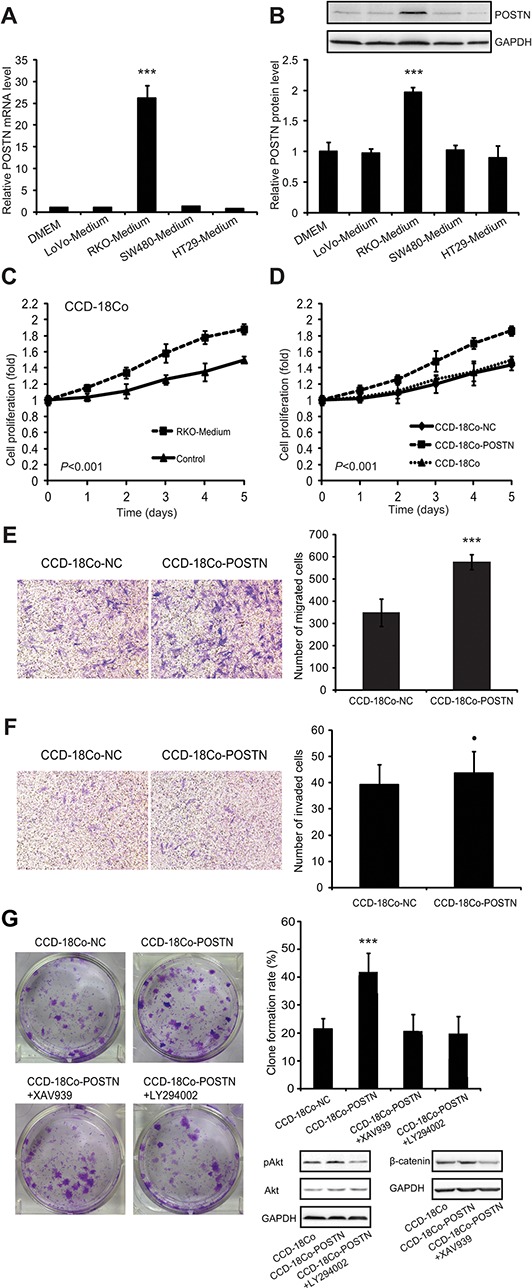
POSTN promoted proliferation of fibroblasts *via* autocrine or paracrine modes of action **A.** the effect of CRC cell conditional media on POSTN mRNA transcription in CCD-18Co colonic fibroblasts, as measured by qRT-PCR. **B.** the effect of CRC cell conditional media on POSTN protein production in CCD-18Co cells by western blot analysis. **C.** proliferation of CCD-18Co cultured with RKO conditional medium and CCD-18Co with normal complete medium. **D.** proliferation of CCD-18Co-POSTN, CCD-18Co-NC, and parental CCD-18Co cells. **E.** migration of CCD-18Co-POSTN and CCD-18Co-NC cells in the Transwell chamber without matrigel. **F.** Invasion of CCD-18Co-POSTN and CCD-18Co-NC cells in the Transwell chamber with matrigel. **G.** anchorage independent growth of CCD-18Co-POSTN and CCD-18Co-NC cells and the effect of the signaling inhibitor LY294002 or XAV939 on POSTON-upregulated anchorage independent growth and expression levels of pAkt and β-catenin in colonic fibroblasts. Abbreviations and marks: CRC, colorectal carcinoma; pAkt, phosphorylation of Akt; qRT-PCR, real-time quantitative RT-PCR; •, *P* > 0.05; ***, *P* < 0.001.

To explore the effect of CRC cells on expression of POSTN in colonic fibroblasts, we cultured parental CCD-18Co cells with conditional media of CRC cell lines SW480, HT29, LoVo, and RKO, respectively. It was found that RKO conditional medium significantly induced the expression of POSTN in CCD-18Co, as verified by qRT-PCR and Western blot analyses (Figure 6A, 6B). Surprisingly, CCD-18Co cultured with RKO conditional medium grew significantly faster than did CCD-18Co cultured routinely (Figure 6C). CCD-18Co-POSTN cells with high POSTN expression also grew faster than did the counterparts with low POSTN expression (CCD-18Co-NC and CCD-18Co) (Figure 6D). Further experimentation indicated that CCD-18Co-POSTN was more migratory but not more invasive than CCD-18Co-NC (Figure 6E, 6F). CCD-18Co-POSTN had a higher clone formation rate than did CCD-18Co-NC, but this effect was counteracted by LY294002 or XAV939. Western blot analyses showed that expression levels of pAkt and β-catenin were higher in CCD-18Co-POSTN than in CCD-18Co cells, however, the levels of pAkt and β-catenin in CCD-18Co-POSTN could be apparently decreased by LY294002 and XAV939, respectively (Figure 6G).

## DISCUSSION

The present study demonstrated that stromal POSTN expression was gradually increased from normal tissues, primary CRC tissues to metastatic CRC tissues (Figure 1), indicating that stromal POSTN expression accumulates consecutively during CRC progression. Importantly, stromal POSTN expression in primary CRC tissues dose-dependently predicted poor postoperative prognoses and had a higher discriminatory performance than did epithelial POSTN expression. Multivariate Cox regression analyses indicated that medium- and high-score stromal POSTN expression, rather than epithelial POSTN expression, predicted unfavorable postoperative prognoses independently in both cohorts (Table 2). Furthermore, medium-score stromal POSTN expression is comparable to the CRC cell-derived gene signature in predicting DFS of the same CRC patients in Shanghai cohort; while medium-and high-score stromal POSTN expression is also comparable to the CRC cell-derived gene signature in predicting DSS in Guangzhou cohort [8]. Thus, stromal POSTN expression in surgically removed tumors should be a powerful and robust prognostic biomarker for CRC, which is worth being clinically translated.

This study revealed that POSTN secreted from gene-transfected colonic fibroblasts or rhPOSTN greatly promoted proliferation, anchorage independent growth, and invasion of CRC cell lines. These effects of POSTN should be realized *via* interacting with its receptors, αv-integrins, on CRC cell lines. αvβ3, αvβ5, and/or α6β4 integrin complex are cell surface receptors of POSTN in CRC, pancreatic cancer, epithelial ovarian carcinoma, esophageal adenocarcinoma, and breast cancer cells [10, 21–23]. We found that external POSTN induced phosphorylation of Akt and the expression of β-catenin in CRC cells and the effects of POSTN on CRC cells could be greatly counteracted *via* targeting PI3K/Akt or Wnt/β-catenin pathway (Figure 4). Based on our findings together with previous publications [9, 10, 14, 17, 18, 21, 22, 24, 25], we believe that POSTN may induce phosphorylation of Akt and focal adhesion kinase (FAK) after binds to its receptors, thus activating PI3K/Akt, NK-κB/STAT3, and ERK pathways, and inducing the expression of multiple downstream genes such as α-smooth muscle actin, fibronectin, chemokines, and transformating growth factor β (TGF-β). POSTN also functions as a driver of EMT and induces expression of matrix metalloproteinase (MMP)-9, -10, and -13, resulting in the degradation of extracellular matrix (ECM), and promoting cancer spread and metastasis [24, 26, 27]. Thus, stromal POSTN whose expression accumulates during CRC progression promotes cancer cell survival, growth, and invasiveness *via* activating oncogenic pathways such as PI3K/Akt and/or Wnt/β-catenin pathways.

We found that stromal POSTN expression in primary CRC tissues dose-dependently predicted an unfavorable prognosis of stage III CRC patients with postoperative chemotherapy (Figure 3). Interestingly, POSTN in cell culture significantly improved the chemo-resistance of CRC cells but this effect was greatly decreased *via* targeting to PI3K/Akt or Wnt/β-catenin pathways (Figure 5). Tumor stem cells are naturally chemoresistant. Molecules that confer the chemoresistence usually increase the “stemness” of cancer cells *via* activating the “stemness”-supportive signaling pathways such as Wnt/β-catenin [28, 29]. Ectopic overexpression of POSTN or recombinant POSTN can induce multipotent stem cell-like properties in human mammary epithelial cells and breast cancer cells [30]. Expression of POSTN is significantly higher in CD133^+^ CRC cells compared to CD133^−^ CRC cells [11]. Wnt/β-catenin signaling pathway often promotes the evolution of the chemo-resistant “stemness” of CRC [28, 29, 31]. Targeting PI3K/Akt or ERK signaling pathway can attenuate the growth of CRC cells or sensitize CRC cell lines to 5-FU [32, 33]. Based on our results and the published data, we hypothesize that POSTN derived from colonic fibroblasts facilitates the evolution of cancer stem cells *via* activating PI3K/Akt and/or Wnt/β-catenin signaling pathways. Our data support that stromal POSTN expression is predictive for chemo-resistance of CRC. Targeting POSTN/αvβ3–6 integrins/PI3K/Akt and/or Wnt/β-catenin pathways should be therapeutic options for metastatic or chemoresistant CRC.

It is well established that chronic inflammation promotes the development of CRC. Some proinflammatory or immunosuppressive molecules including TGF-β1, 2, and 3, vascular endothelial growth factor (VEGF), interleukin (IL)-3, 4, 6, and 13 can induce the expression of POSTN in a cell-specific context [10]. In this study, we found that conditional medium of RKO could induce POSTN production in fibroblasts, possibly because RKO secretes immunosuppressive factors such as TGF-β1, VEGF, IL-4, IL-6, IL-10, and prostaglandin E2 [34]. These CRC cell-derived factors can induce the expression of POSTN in colonic fibroblasts and autocrine POSTN in turn promoted proliferation, migration, and anchorage independent growth of colonic fibroblasts (Figure 6). Cancer cells can induce fibroblast-mediated accumulation of stromal POSTN and POSTN derived from cancer stem cells can recruit M2 tumor-associated macrophages and promotes malignant growth [35, 36], indicating that POSTN bridges cancer cells and cancer-supportive stromal cells. Like other cancer biomarkers such as α-fetoprotein, POSTN is actively expressed in a specific temporal and spatial pattern during embryogenesis, silenced after birth, and re-expressed in response to mechanical stress or carcinogenesis [9]. These evidences indicate that stromal POSTN may be re-expressed by proinflammatory factors in chronic tumor-supportive inflammation, and facilitate cancer evolution and development *via* creating a cancer-supportive niche.

In summary, stromal POSTN expression in primary tumor tissues independently predicted an unfavorable prognosis of CRC patients after the adjustment with covariates including TNM stage and postoperative chemotherapy. Stromal POSTN expression also predicted a poor prognosis of CRC patients with postoperative chemotherapy dose-dependently. Both of POSTN derived from colonic fibroblasts and rhPOSTN significantly promoted proliferation, anchorage independent growth, invasion, and chemo-resistance of CRC cells; however these effects were greatly counteracted *via* targeting to PI3K/Akt or Wnt/β-catenin pathways. CRC cell line RKO-secreted factors can induce the production of POSTN in colonic fibroblasts and POSTN in turn promoted proliferation, migration, and anchorage independent growth of colonic fibroblasts. Thus, stromal POSTN expression is prognostic and predictive for CRC, which is worth being clinically translated. POSTN may facilitate the evolution and development of CRC *via* creating a cancer-supportive niche. Targeting POSTN-induced signaling pathways should be therapeutic options for metastatic or chemoresistant CRC.

## MATERIALS AND METHODS

### Patients

Pathologically proven formalin-fixed paraffin-embedded (FFPE) specimens of 1098 CRC patients were enrolled in this study. Of these, 755 received curative surgery in the 1^st^ Affiliated Hospital of Second Military Medical University (Shanghai, China) between January 2001 and December 2009 and 343 received curative surgery in the Sixth Affiliated Hospital of Sun Yet-Sen University (Guangzhou, China) between January 2000 and January 2006. In addition, we also included FFPE specimens of surgically removed adjacent mucosa from 37 CRC patients and surgically removed metastatic CRC from 21 CRC patients. TNM staging was reclassified according to the American Joint Committee on Cancer (AJCC) staging system (seventh edition). Some of them, especially stage III CRC patients received a standard postoperative chemotherapy (FOLFOX regimen). Baseline information of each specimen donor in Shanghai cohort was summarized in Table 1. Follow-up exam of 1025 patients with stage I–III CRC were carried out as previously reported [8]. DFS and DSS were defined as previously described [8]. This study was approved by the institutional review boards of The 1^st^ Affiliated Hospital of Second Military Medical University and The Sixth Affiliated Hospital of Sun Yet-Sen University. Informed consent has been obtained.

### IHC

TMAs were developed as previously described [8]. IHC was carried out in Pathology Core Laboratory of the 1^st^ Affiliated Hospital of Second Military Medical University. Rabbit polyclonal antibodies to human POSTN (1:500, ab14041, Abcam, Cambridge, UK) were used for IHC according to protocols provided by the manufacturers. The specificity and intratumoural heterogeneity of this antibody has been verified in our previous study [8]. Scores were independently assessed by the first 4 authors and then verified by two pathologists (YY and YD). Briefly, intensity of immunostaining in stromal cells or epithelial cancer cells in tumor specimens was graded as 0 (negative), 1 (weak), 2 (moderate) and 3 (strong); staining extent was graded as 0 (0%–4%), 1 (5%–24%), 2 (25%–49%), 3 (50%–74%) and 4 (>75%). Values of the intensity and the extent were multiplied as an immunoreactive score. POSTN expression was graded as low (scores, 0–4), medium (scores, 6–8) and high (scores, 9–12). Disagreements among the researchers were resolved by consensus.

### Cell culture

Human colonic fibroblast cell line (CCD-18Co) and 4 human CRC cell lines (SW480, HT29, LoVo, and RKO) were purchased from American Type Culture Collection (Manassas, VA). All cell lines were maintained in Dulbecco's Modified Eagle Medium (GIBCO, Grand Island, NY, USA) supplemented with 10% heat-inactivated fetal bovine serum (FBS) (GIBCO), 100 U/mL penicillin, and 100 μg/mL streptomycin in a 5% CO_2_ humidified atmosphere.

### Vector construction, transfection, and generation of POSTN-producing colonic fibroblasts

A full-length of human POSTN cDNA clone (CDS-h13016-2) was purchased from Axybio (Changsha, China), released by SalI and EcoRI digestion, and inserted into pLenO-GTP lentiviral vector (Invabio, Shanghai, China). The resulting pLenO-GTP-POSTN lentiviral construct was verified by direct DNA sequencing. The empty pLenO-GTP lentiviral vector served as a negative control, named pLenO-GTP-NC. The two constructs pLenO-GTP-POSTN and pLenO-GTP-NC were transfected into 293T cells to propagate lentiviruses, respectively. CCD-18Co cells were incubated with lentiviruses containing pLenO-GTP-POSTN or pLenO-GTP-NC with 4 mg/ml polybrene (Sigma-Aldrich, St. Louis) for 96 hrs, and then selected in the presence of 3.5 mg/ml puromycin (Sigma-Aldrich) for 7 d. Finally, we selected a CCD-18Co-POSTN cell line with stable POSTN expression and a control CCD-18Co-NC cell line.

### qRT-PCR

qRT-PCR was firstly applied to measure levels of POSTN mRNA. The primers for the examination of POSTN mRNA were 5′-GCACTCTGGGCATCGTGGGA-3′ (forward) and 5′-AATCCAAGTTGTCCCAAGCC-3′ (reverse), for GAPDH (an internal control) were 5′-AAATCCCATCACCATCTTCC-3′ (forward) and 5′-TCCACCACCCTGTTGCTGTA-3′ (reverse). qRT-PCR was performed as we previously described [37].

### Western blot

Rabbit monoclonal antibody (mAb) to human POSTN (1:1000, ab172615, Abcam, UK), mouse mAb to human Phospho-Akt (Ser473) (587F11) (1:1000, 4051, Cell Signaling Technology, Beverly, MA), rabbit polyclonal antibody (pAb) to human Akt (1:1000, 9372, Cell Signaling Technology), rabbit mAb to human β-catenin (1:1000, 8480, Cell Signaling Technology) and rabbit pAb to human GAPDH (1:1000, AP0063, Bioworld Technology) were applied for Western blot analysis. Signal intensity of each band was quantified using Genetools software (version 4.02, Cambridge, UK). Relative expression was calculated as that signal intensity of POSTN divided by signal intensity of GAPDH in each lane as previously described [38].

### Proliferation, invasion, and anchorage independent growth assays of CRC cells

CCD-18Co-POSTN and CCD-18Co-NC were cultured with FBS-free DMEM for 48 h, respectively. The supernatants were aseptically filtered and mixed with 5% FBS as conditional media. SW480 and HT29 were cultured with 100% conditional media of colonic fibroblasts, complete medium with 100 ng/mL rhPOSTN (10299-H08H, Sino Biological, Beijing, China), and complete medium alone, respectively. PI3K/Akt kinase inhibitor, LY294002 (Selleck, Shanghai, China) at the final concentration of 10 μM or β-catenin-specific inhibitor, XAV939 (Selleck, Shanghai, China) at the same concentration was added into cell cultures to assess if the effect of rhPOSTN was mediated by these signaling pathways. The same amount of Dimethyl Sulphoxide, dissolvant of the inhibitors, was also added to cell cultures with or without rhPOSTN. Cell proliferation assay was examined by using cell counting kit-8 (Beyotime, China) according to the manufacture instruction. Cell invasion assay was performed using 8-mm pore size 24-well tissue culture Transwell plates (Corning, NY) as previously described [39], with the exception that 100 ng/mL rhPOSTN was added into medium in both chambers in the experimental group. Anchorage independent growth potential was evaluated by double-layered soft agarose culture system. Diluted SW480 and HT29 cells were seeded into 6-cm culture dishes (1000 cells/dish) and incubated for 14 to 21 days, respectively. Cell clones in the dishes were fixed with 4% paraformaldehyde for 15 min and stained with 0.1% crystal violet for 10 min. The number of stained clones containing >50 cells was counted as survivors, and clone formation rate was calculated with the formula: the number of survivors/1000 × 100%. All examinations were performed in triplicate.

### Chemo-resistance of CRC cells to 5-FU

SW480 and HT29 were seeded in 96-well plates (5 × 10^3^/100 μL per well) and cultured with conditional medium of CCD-18Co-NC, the medium of CCD-18Co-POSTN, 100 ng/mL rhPOSTON, and 100 ng/mL rhPOSTON plus 10 μM each of the 2 signaling inhibitors, respectively. The cells allowed to grow for 24 h and then treated with 5-FU at various concentrations (0, 5, 10, 25, 50, and 100 μg/mL) for 36 h. Number of viable cells was measured by using cell counting kit-8 assay. Then, inhibition of viable cells was calculated according to the inhibition rate, as previously described [7, 40].

### Effects of CRC-derived factors on the production of POSTN in fibroblasts and autocrine POSTN on the growth, migration, invasion, and anchorage independent growth of fibroblasts

LoVo, SW480, HT29, and RKO were cultured with serum-free DMEM for 48 h. The supernatants of the cells were sterile filtered and mixed with FBS at the final concentration of 10% to make CRC conditional medium, respectively. The conditional medium of each CRC cell line was applied to culture CCD-18Co cells. After 7 days' culture, CCD-18Co cells were harvested and subjected to exam the expression of POSTN by qRT-PCR and Western blot. The growth of CCD-18Co in response to RKO conditional medium as well as cell proliferation, invasion, and anchorage independent growth assays of CCD-18Co-NC and CCD-18Co-POSTN were examined as described above. Cell migration assay was carried out as the invasion assay except that the bottom of the culture insert was not coated with Matrigel. The two inhibitors targeting to signaling pathways were added in cell proliferation and anchorage independent growth assays of CCD-18Co-POSTN. Western blot was applied to exam the effects of the inhibitors on the expression of corresponding signaling proteins in colonic fibroblasts.

### Statistical analysis

Kruskal-Wallis test and Mann-Whitney *U* test was used to evaluate the immunostaining score of stromal POSTN expression in adjacent mucosa tissues, primary CRC tissues, and metastatic CRC tissues of CRC patients. Kaplan-Meier analysis with log-rank test was used to estimate DFS of CRC patients in Shanghai cohort and DSS of CRC patients in Shanghai and Guangzhou cohorts. Multivariate Cox hazard proportion models were applied to determine contributions of stromal POSTN expression and epithelial POSTN expression to the survivals, adjusted for age, gender, disease location, TNM stage, tumor differentiation grade, lymph nodes examined, serum CEA, serum CA19-9, and adjuvant chemotherapy. All statistical tests were two-sided and done with Statistical Program for Social Sciences (SPSS 16.0 for Windows, SPSS, Chicago, IL). *P* < 0.05 was considered as statistically significant.
